# Normative values of cardiorespiratory fitness in Croatian children and adolescents

**DOI:** 10.1371/journal.pone.0284410

**Published:** 2023-04-24

**Authors:** Peter Sagat, Lovro Štefan, Vilko Petrić, Vesna Štemberger, Iva Blažević

**Affiliations:** 1 Department of Health and Physical Education, Prince Sultan University, Riyadh, Saudi Arabia; 2 Department of General and Applied Kinesiology, Faculty of Kinesiology, University of Zagreb, Zagreb, Croatia; 3 Department of Sport Motorics and Methodology in Kinanthropology, Faculty of Sports Studies, Masaryk University, Brno, Czech Republic; 4 Department of Educational Studies, Faculty of Teacher Education, University of Rijeka, Rijeka, Croatia; 5 Department of Primary Teacher Education, Faculty of Education, University of Ljubljana, Ljubljana, Slovenia; 6 Department of Primary Teacher Education, Faculty of Educational Science, University of Pula, Pula, Croatia; Bangor University, UNITED KINGDOM

## Abstract

Although defining normative values of cardiorespiratory fitness have been the topic of many Western societies, little evidence has been provided for less developed countries like Croatia. Since cardiorespiratory fitness rapidly declines in Croatian children and adolescents, the newly established normative values would help health-related professionals and physical education teachers to detect ‘talented’ groups and direct them towards sport and ‘risky’ groups for planning special interventions. Therefore, the main purpose of the study was to determine normative reference values of cardiorespiratory fitness. A total of 1,612 children and adolescents aged 7–14 years (mean±SD; age 9.7±2.4 years; stature 151.0±17.6 cm; body mass 45.1±19.1 kg; 52.5% girls) participated in this cross-sectional study. Cardiorespiratory fitness was assessed by the Maximal multistage 20-m shuttle run test and the performance was expressed as the number of stages. Maximal oxygen uptake (VO_2_max) was estimated by equations. Smoothed percentile curves were calculated. Boys outperformed girls in the maximal number of levels achieved after the 20-m shuttle run test and in the VO_2_max values at each age category. In boys, a gradually higher level of performance between ages 11 and 14 was observed, while in girls the values started to rise after the age of 8. Our study provides one of the first sex- and age-specific normative values for cardiorespiratory fitness assessed by the 20-m shuttle run test in Croatian children and adolescents.

## Introduction

Physical fitness is a multidimensional construct often defined as ‘the capacity to perform physical activity and refers to a full range of physiological and psychological qualities’ [1]. The components of physical fitness are: 1) cardiorespiratory fitness, 2) muscular fitness and speed/agility [1]. As an important indicator of health in youth, cardiorespiratory fitness has the ability to transport oxygen and macro- and micro- nutrients for optimal muscle activity during exercise [2, 3]. Lower levels of cardiorespiratory fitness have been associated with higher cardiovascular and metabolic diseases, including higher abdominal obesity, blood pressure, fasting blood glucose and triglycerides, irrespective of socio-demographic factors, diet and physical activity [4–6]. Also, it has been shown that cardiorespiratory fitness tracks moderately well from childhood to adulthood [7–9], suggesting that acquiring high cardiorespiratory fitness is a protective factor against the development of chronic diseases later in life.

The level of cardiorespiratory fitness has been mostly assessed in a field-based setting [5, 10, 11]. The most common field-based test to determine the level of cardiorespiratory endurance is the 20-m shuttle run test [10, 11]. Although the World Health Organization has recommended graded exercise testing to exhaustion as the ‘gold standard’ measurement technique [12], the 20-m shuttle run test has shown strong-to-very strong reliability and moderate-to-strong validity properties [13]. Moreover, the utility of the test lies in its low cost, flexibility of testing locations and measuring individuals simultaneously [5, 10–13].

In order to extend the applicability of the 20-m shuttle run test, it is necessary to develop international norms, based on different geographical and demographical regions. To date, several normative reference values established from the 20-m shuttle run test have been published to identify children and adolescents with both low and high cardiorespiratory fitness [14–26]. Most of them have come from North America [14–16], Europe [17–20], China [21–23], Japan [24], the Philippines [25] and South Korea [26]. Despite the effort to provide the normative reference values for the 20-m shuttle run test, there is a scarcity of such standards for children and adolescents from low-middle income countries going through a period of socioeconomic, epidemiologic and nutritional transitions. In Croatia, no population-based studies have been conducted to assess the level of cardiorespiratory fitness in Croatian youth. Although the data have been analyzed from 1999 to 2014, a recent study conducted in Croatian youth has shown a negative trend in cardiorespiratory fitness, meaning that children and adolescents measured in earlier years performed better, compared to their counterparts measured in later years [27]. This would further imply the overall decline in cardiorespiratory fitness, being predominately observed in recent years. The latest study from 34 countries and nearly 8 million data results has presented Croatia as a country with intermediate cardiorespiratory fitness level, based on the mean centile of the 20-m shuttle run test [28]. However, the ranking was strictly based on a relatively small sample size (*N* = 596) and narrow age range (15–18 years), while the data from primary school children have not been included. Since the level of fitness starts to shape primarily in childhood [1], it is necessary to establish reference data in that age range. By defining the normative reference values, health-related professionals and physical education teachers would be given an insight into the current health status and the detection of those individuals with both low and high cardiorespiratory fitness. Thus, the findings would have a 2-fold advantage: (i) to detect children, who are at increased risk of having low cardiorespiratory fitness and to implement strategies for promotion of health-related fitness behaviors; and (ii) to identify children with high cardiorespiratory fitness, who may be directed towards higher levels of physical activity [17].

Therefore, the main purpose of the study was to determine normative reference values of cardiorespiratory fitness assessed by the 20-m shuttle run test in a sample of 7- to 14-year old school-aged children and adolescents from Croatia. By developing growth charts, we would be able to conduct comparisons with previously established international norms.

## Materials and methods

### Study participants and testing procedure

In this observational, cross-sectional study, we relied on a stratified two-stage random sampling procedures to select a representative sample of urban school-going children 7 to 14 years of age. Sample size calculations based on 16 groups (boys and girls in each age category) suggested that a total sample of 496 individuals would be needed to detect small effects (*f*_2_ = 0.20) with a statistical power of 0.80 and alpha set at 0.05. Since the purpose of the study was to establish generalizable reference data for the 20-m shuttle run, we had to increase our targeted sample size. Assuming a total population of children in the city of Zagreb of 65,500 and with a margin of error to be set at 2.5%, the estimated sample size was set at 1,600. In the first stage, of ≈150 primary schools located in the city of Zagreb, we randomly recruited 16 schools (14 public and 2 private schools), with each school having equal probability of selection. Of them, 12 schools (10 public and 2 private schools) had agreed to take part in the study. During the second stage of selection, one class (a, b or c class) was randomly selected to represent one age group within each school (boys and girls aged 7 to 14 years). Initially, 1,950 children were selected. The following inclusion criteria were applied: 1) being without locomotor or mental problems, 2) regularly attending physical education classes and 3) a total completition of the 20-m shuttle run test. Of 1,950 children, 338 were not tested for the 20-m shuttle run test or were absent from school during the testing day. The analyses were based on 1,612 school aged children aged 7–14-years old (response rate = 82.7%, 52.5% girls). Testing procedures were followed and standardized by previous studies [29], to minimize the effects of environmental factors and to avoid fatigue. The 20-m shuttle run test was measured from September to October in each school. All physical education teachers were instructed about the testing procedure for undertaking the 20-m shuttle run test. During the testing, children were told to wear a light T-shirt, shorts and training shoes. All procedures performed in this study were anonymous and were conducted according to Declaration of Helsinki. The study was approved by the Faculty of Kinesiology, University of Zagreb, Croatia. The written informed consent voluntarily was signed by the participants, participants’ parents or their guardians to have data from their records used in research.

### The 20-m shuttle run test

To assess cardiorespiratory fitness, the 20-m shuttle run test was applied [17, 30]. More detailed information of conducting the test has been previously described [17, 30]. In brief, parallel lines 20 m apart were constructed and children had to run back and forth, following the pace of an audio signal that began at a speed of 8.5 km/h and increased by 0.5 km/h at 1-minute intervals. The complete testing procedure was undertaken indoors. The maximal number of levels (#) achieved after the 20-m shuttle run test was written as the final score. To express maximal oxygen uptake (VO_2_max), we used the equations previously described by Léger *et al*. [31]. A high correlation between estimated and objectively measured VO_2_max has been reported previously (*r* = 0.71, *p*<0.001) [31].

### Data analysis

Basic descriptive statistics of the study participants are presented as mean and standard deviation (SD). To determine the normality of distributions, we ran the Kolmogorov–Smirnov test. Sex and age differences were calculated by using analysis of variance (ANOVA) with post hoc comparison test between the groups. For the maximal number of levels and VO_2_max, we determined the 5th, 10th, 25th, 50th, 75th, 90th and 95th sex- and age-specific percentile values by using the Lambda (L), Mu (M) and Sigma (S) method. In this method, the optimal power to obtain normality is summarized by the skewness (L), median (M) and coefficient of variation (S). For the purpose of this study, we fitted smooth centile curves to develop sex-and age-specific 20-m SRT reference standards of Croatian children and adolescents by combining the changing distribution of three sex- and age-specific curves [32]. In the next step, all three curves (L, M and S) are summarized based on the power of age-specific Box–Cox power transformations for normalizing the data. A power transformation is then applied to normalize the data and to remove the skewness [32]. The estimation of these parameters as a function uses a penalized maximum likelihood approach where the penalty term is com- posed of integrals over the squared second derivatives expressing the smoothness of the curves. The curves can be fitted as cubic splines using non-linear regression, and the extent of smoothing required can be expressed in terms of smoothing parameters or equivalent degrees of freedom using penalized likelihood [32]. All analyses were performed in Statistical Packages for Social Sciences version 24. (SPSS Inc., Chicago, Illinois, USA) and the significance was set at *p*<0.05.

## Results

Basic descriptive statistics of the study participants are presented in Table 1.

**Table 1:** Basic descriptive statistics of the study participants (*N* = 1,612).

Study variables	Total (*N* = 1,612)	Boys (*N* = 766)	Girls (*N* = 846)
	mean (SD)	mean (SD)	mean (SD)
Age (years)	9.7 (2.4)	9.8 (2.4)	9.6 (2.3)
Height (cm)	151.0 (17.6)	152.0 (19.4)	150.2 (15.7)
Weight (kg)	45.1 (19.1)	46.5 (13.3)	43.9 (14.0)
Body-mass index (kg/m^2^)	19.0 (3.5)	19.2 (3.7)	18.9 (3.3)
Waist circumference (cm)	65.4 (9.5)	67.1 (10.0)	64.0 (8.8)
Waist-to-height ratio	0.43 (0.05)	0.44 (0.05)	0.43 (0.05)
20-m shuttle run (level)	4.3 (1.9)	4.9 (2.2)	3.9 (1.4)
VO_2_max (mlO_2_/kg/min)	45.7 (5.2)	47.8 (5.5)	43.8 (4.1)

*p*<0.05.

Sex- and age- specific descriptive statistics for the maximal number of levels achieved after the 20-m shuttle run test and the VO_2_max values are presented in Table 2. In specific, boys outperformed girls in the maximal number of levels completed after the 20-m shuttle run test (*F*_1,15_ = 102.07, *p* < 0.001) and in the VO_2_max (*F*_1,15_ = 218.23, *p* < 0.001). Age-specific analyses show that values in the maximal number of levels remain similar till the age of 10, after which they start to increase till the age of 14 (*F*_1,15_ = 44.62, *p* < 0.001). For the VO_2_max, the trajectory seems to decline between the ages 7 and 11, after which the value starts to increase (*F*_1,15_ = 3.95, *p* < 0.001). Analyses regarding the interaction effect between sex and age show that boys in the specific age group achieve greater values in the maximal number of levels achieved after the 20-m shuttle run test (*F*_1,15_ = 4.86, *p* < 0.001) and in the VO_2_max (*F*_1,15_ = 4.06, *p* < 0.001), compared to girls in the same age group.

**Table 2:** Sex- and age- specific data for the maximal number of levels and the VO_2_max.

	Level (#)	VO_2_max (mL/kg/min)
**Boys**	**Mean (SD)**	**Mean (SD)**
**7**	4.8 (2.2)	48.8 (4.8)
**8**	3.3 (1.1)	47.0 (3.7)
**9**	4.6 (1.8)	48.2 (3.9)
**10**	4.2 (1.6)	46.9 (5.2)
**11**	4.5 (1.8)	45.7 (6.5)
**12**	5.2 (2.3)	46.5 (7.2)
**13**	6.2 (2.0)	48.7 (6.2)
**14**	6.9 (2.4)	49.2 (6.3)
**Girls**		
**7**	3.6 (1.2)	44.5 (3.6)
**8**	3.1 (0.9)	44.5 (3.8)
**9**	3.4 (1.1)	43.3 (3.9)
**10**	3.7 (1.2)	43.8 (4.7)
**11**	4.2 (1.2)	43.7 (4.4)
**12**	4.5 (1.5)	43.6 (4.2)
**13**	4.9 (1.2)	43.6 (3.9)
**14**	5.1 (1.6)	42.6 (5.1)

**Fig 1:**
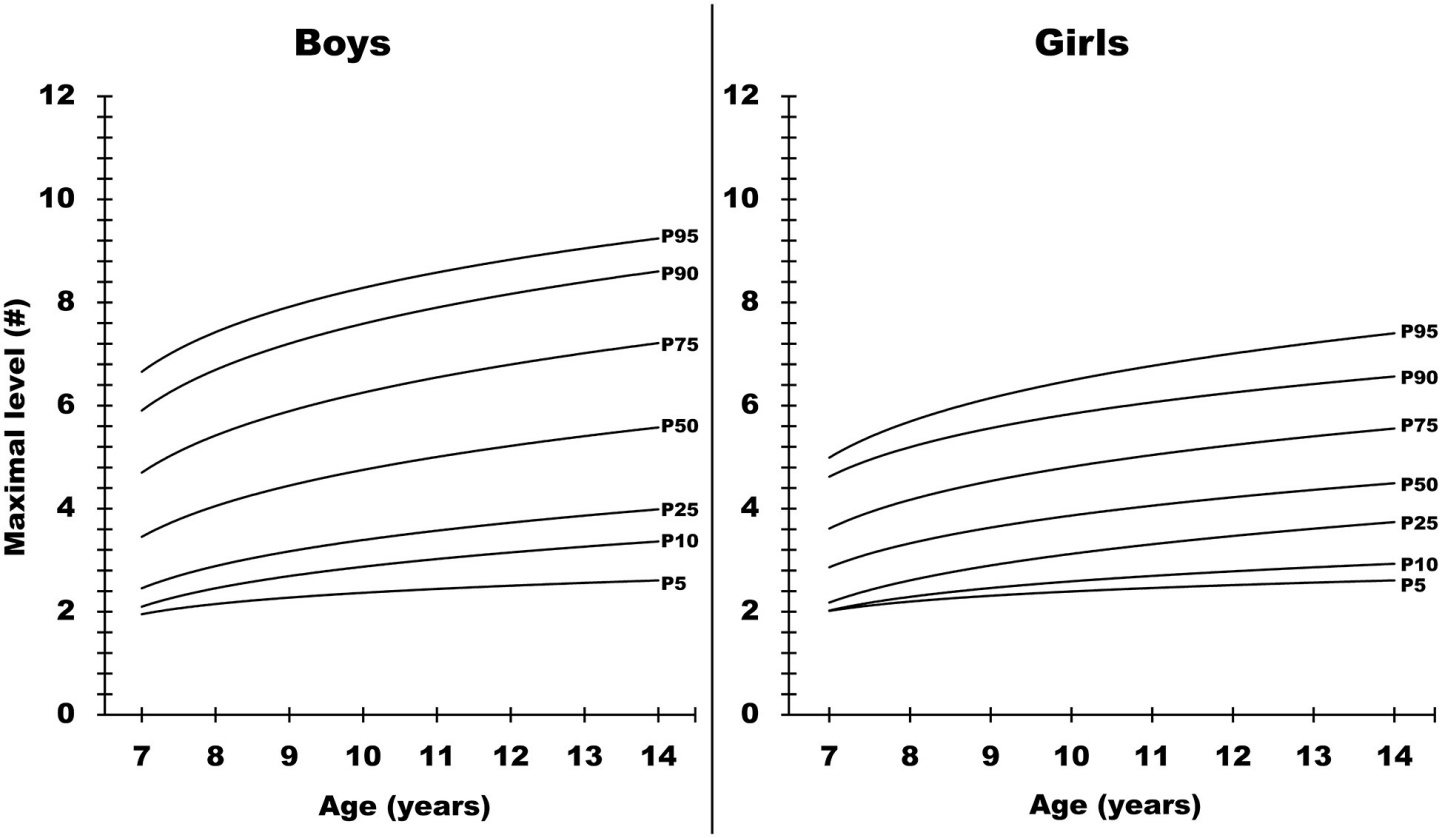
Graphical normative reference data for the maximal number of levels achieved after the 20-m shuttle run test, according to sex and age.

**Fig 2:**
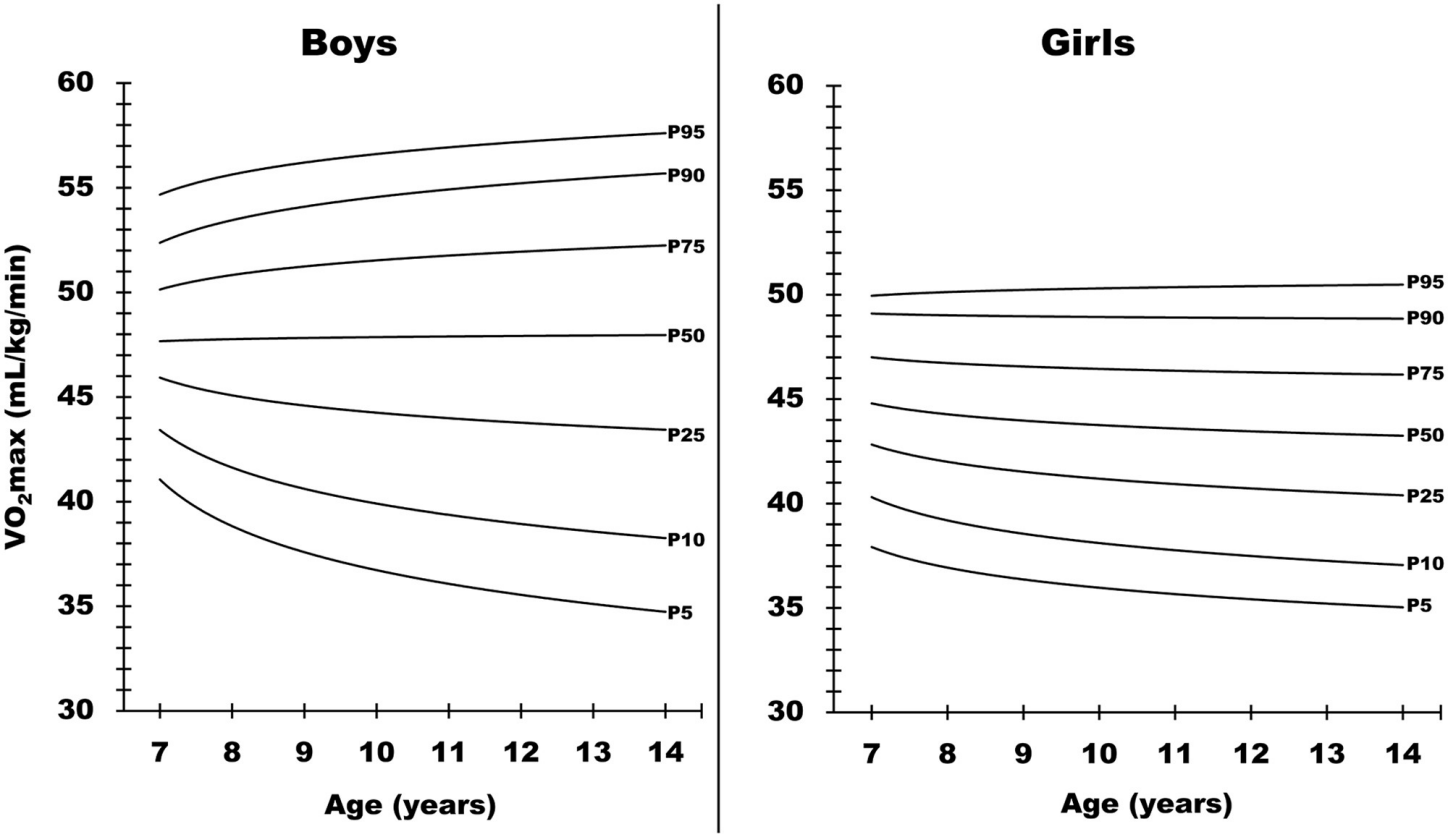
Graphical normative reference data for the VO_2_max (ml/kg/min) derived from the equation, according to sex and age.

Tables 3 and 4 present numerical data approximating the 5^th^, 10^th^, 25^th^, 50^th^, 75^th^, 90^th^ and 95^th^ centiles for the maximal number of levels achieved after the 20-m shuttle run test and the VO_2_max. Equivalent graphical values are available in Figs 1 and 2.

**Table 3:** Sex- and age-specific normative reference values for the maximal number of levels achieved after the 20-m shuttle run test (*N* = 1,612).

Sex	Age	N	P5	P10	P25	P50	P75	P90	P95
Boys	7	240	2.10	2.30	3.10	4.30	6.30	8.00	8.55
	8	74	2.10	2.20	2.48	3.20	3.80	4.66	5.53
	9	160	2.30	2.60	3.10	4.45	6.10	7.20	8.01
	10	70	2.10	2.20	2.80	4.30	5.20	6.54	7.14
	11	93	2.20	2.30	2.80	4.30	5.80	7.10	7.90
	12	70	2.32	2.64	3.30	5.10	7.10	8.52	9.26
	13	71	3.04	3.20	4.80	6.10	8.10	9.12	9.31
	14	69	2.79	3.30	5.28	6.60	8.33	10.39	11.15
Girls	7	200	2.20	2.30	2.60	3.30	4.20	5.51	6.30
	8	87	2.10	2.10	2.33	3.10	3.75	4.37	4.61
	9	107	2.14	2.30	2.60	3.20	4.10	5.10	5.20
	10	73	2.20	2.30	2.70	3.30	4.25	5.50	6.20
	11	82	2.50	2.65	3.20	4.20	5.05	5.75	6.45
	12	67	2.36	2.70	3.40	4.30	5.50	6.70	7.53
	13	75	3.10	3.24	4.10	4.90	5.60	6.62	7.46
	14	74	2.55	3.13	4.20	4.70	6.10	7.22	8.46

**Table 4:** Sex- and age-specific normative reference values for the VO_2_max (ml/kg/min; *N* = 1,612).

Sex	Age	N	P5	P10	P25	P50	P75	P90	P95
Boys	7	240	39.73	42.84	48.68	52.18	54.57	56.65	39.73
	8	74	40.01	42.6	46.88	48.88	51.46	54.37	40.01
	9	160	40.84	42.44	48.11	50.65	53.89	55.21	40.84
	10	70	35.86	40.13	47.68	50.30	53.54	54.93	35.86
	11	93	34.91	36.94	46.22	51.35	52.56	55.62	34.91
	12	70	32.38	36.12	46.68	51.9	55.48	58.71	32.38
	13	71	36.23	38.78	49.06	53.42	56.78	57.3	36.23
	14	69	36.09	41.20	49.62	53.24	57.69	59.64	36.09
Girls	7	200	37.63	40.28	44.51	46.87	49.24	50.34	37.63
	8	87	37.33	39.06	45.02	47.05	48.67	49.36	37.33
	9	107	36.82	39.01	43.45	45.64	48.28	49.61	36.82
	10	73	33.97	37.52	43.67	47.09	49.89	51.43	33.97
	11	82	36.46	37.59	43.64	46.93	49.14	50.79	36.46
	12	67	36.85	38.17	43.57	46.57	49.56	50.4	36.85
	13	75	37.03	37.9	43.95	45.82	48.04	49.61	37.03
	14	74	32.65	36.22	42.64	45.83	48.72	50.78	32.65

## Discussion

The main purpose of the study was to determine normative reference values of cardiorespiratory fitness assessed by the 20-m shuttle run test in a sample of 7- to 14-year-old school-aged children and adolescents from Croatia. The main findings of the study are: 1) boys outperformed girls in the maximal number of levels achieved after the 20-m shuttle run test and in the VO_2_max; 2) older boys and girls had better results in the maximal number of levels achieved after the 20-m shuttle run test and in the VO_2_max, compared to their younger counterparts; and 3) boys had higher performance than girls at each age category.

This is the first population-based study aiming to establish normative reference values for cardiorespiratory fitness in Croatian children and adolescents. The findings of the study are in line and can be compared with previous research, due to the same methodology and principles being used to create percentile charts for the VO_2_max [14–26]. In a most comprehensive and up-to-date cross-sectional normative reference study by Tomkinson *et al*. [17], boys outperformed girls and experienced larger age-related changes, due to typical maturational changes in aerobic test performance [33]. Previous evidence has suggested that such changes occur due to different physiological factors associated with puberty [33]. It has been suggested that the increase in VO_2_max for boys is usually explained by greater muscle mass and higher hemoglobin concentration in puberty [33], while no positive effect between puberty and aerobic power was observed in girls [34]. Another mechanism for sex-related differences may be due to more pronounced activities of moderate and vigorous intensities performed by boys, while girls engage in lower intensity physical activity [35]. In boys, cardiorespiratory performance steadily increased, but was the steepest between the ages 11 and 14, which is similar to one other study [36]. The level of VO_2_max obtained in our study was similar to other findings from Portuguese [18] and Norwegian [35] adolescents. Although special interventions aiming to increase the level of physical fitness have shown a relatively small effect to date, it has been highlighted that school environment serves as the most potent setting for behavioral interventions [37].

Although biological aspects explaining the differences between boys and girls in the cardiorespiratory fitness have been well-described [17], it is necessary to put into perspective our newly reference data for Croatia and compare them with international norms [17, 28]. In a systematic review by Tomkinson *et al*. [17], the 50^th^ percentile for the maximal number of levels ranged from 4.07 to 5.93 in boys and from 3.43 to 3.78 in girls. Similar findings are observed for the VO_2_max, where the corresponding values for the 50^th^ percentile are 48.1 (9-year-olds) to 44.6 mL/kg/min (14-year-olds) in boys and 46.7 to 38.8 mL/kg/min in girls. Our findings showed that children and adolescents in a certain age group performed better, compared to international norms [17]. This has been confirmed in the most recent European study, which positions Croatia as having “intermediate” level of cardiorespiratory fitness (16^th^ place out of 34 countries studied) with percentile ranks of 58.1 and 52.5 for boys and girls, respectively [28]. However, a study by Tomkinson *et al*. [17] used the worldwide data from 50 countries and 9–17-year-olds, compared to Ortega *et al*. [28], who based reference data on 34 European countries. Nevertheless, our findings suggest that both boys and girls surpass the median value of the 20-m shuttle run test on European [28] and worldwide levels [17], pointing out that interventions should be directed towards enhancing or even maintaining cardiorespiratory fitness above the median value. Although we observed that boys and girls in this study had the VO_2_max values above the international norms [17, 28], a most recent study has shown that Croatian youth is facing with the pandemic of being overweight and obesity, being 35.9% for children, placing Croatia at the top of ‘risk countries’ in Europe [38]. Since most of so called ‘risk factors’ for future disease development start to occur in childhood and adolescence [29], the importance of our data is multi-fold for future practical utility and implication. By testing and monitoring cardiorespiratory fitness at a young age, policy makers would have an insight into functional fitness of children at individual and group level. It has been-well known that higher levels of cardiorespiratory fitness in childhood and adolescence have beneficial health-related outcomes in adulthood [1, 39], pointing out that annual prevalence of cardiorespiratory fitness should be part of systematic examinations. Unfortunately, most health-related professionals do not have the time to measure such a construct. Since measuring and tracking of physical fitness is part of physical education curricula in many countries, pediatrician’s medical records should be linked to physical fitness databases to create an individual’s health profile. Although we presented the findings for the 20-m shuttle run as a standard field-based test for assessing cardiorespiratory fitness [17, 28], such construct is not commonly used in Croatian educational system. Therefore, testing methodology should be standardized across the countries for comparable data and creating national and international standards. Second, participants categorized in the lowest percentiles should be a target group for special interventions aiming to enhance the level of cardiorespiratory fitness. School-based environment may be a helpful tool because research suggests that interventions implemented within a school setting moderately increase physical activity and cardiorespiratory fitness [40]. Finally, baseline results can be easily remembered and tracked over time.

This study is not without limitations. The level of cardiorespiratory fitness in growing children and adolescents should be obtained from longitudinal studies that give the possibility to assess natural changes in individual growth and development [30]. Along with that, biological maturation, like peak height velocity was not assessed in this study, diminishing the range of variability between individuals of the same chronological age during childhood and adolescence. Also, our findings must be interpreted with caution, since we did not collect the data at country-level, limiting its generalizability.

## Conclusions

Based on the 20-m shuttle run test, the current study provided sex- and age-specific normative reference values for the maximal number of levels and estimated VO_2_max in 7-to-14-year old children and adolescents. This data can help in identifying children and adolescents with high cardiorespiratory fitness and their recruitment towards sporting and athletic development programs. On the other hand, individuals with low cardiorespiratory fitness should be a target group for special intervention and policies to promote health physical fitness behaviors.

## Supporting information

S1 Data (XLSX)
